# Clinical performances of the EuroSCORE II in a single-centre, contemporary cardiac surgical cohort

**DOI:** 10.1186/1749-8090-10-S1-A59

**Published:** 2015-12-16

**Authors:** Dusko Nezic, Tatjana Spasic, Slobodan Micovic, Dragana Kosevic, Milijana Balevic, Ivana Petrovic, Borzanovic Milorad

**Affiliations:** 1 Department of Cardiac Surgery, "Dedinje" Cardiovascular Institute, 11000 Belgrade, Serbia; 2 Department of Cardiology, "Dedinje" Cardiovascular Institute, 11000 Belgrade, Serbia

## Background/Introduction

Risk adjusted perioperative mortality rate following cardiac surgery has been widely used as an indicator of quality of care as well as for comparison of outcomes among institutions and surgeons.

## Aims/Objectives

We aimed to compare and validate the original EuroSCORE risk stratification models with renewed EuroSCORE II model in a contemporary cardiac surgical practice at our institution.

## Method

The additive, logistic and EuroSCORE II data on 1864 consecutive patients undergoing adult cardiac surgery during 2012, were stored in the institutional database. Discriminative power of the EuroSCORE models was tested by calculating the area under the receiver operating characteristic curve (AUC). The calibration of the models were assessed by the Hosmer-Lemeshow statistics, and with the observed to expected mortality ratio. Patients with EuroSCORE II values of 0.5-2.49; 2.5-5.99%; 6.0-9.99%; and ≥ 10% were defined to be at low, moderate, high and very high perioperative risk, respectively

## Results

Excellent discriminatory abilities of all EuroSCORE models were confirmed (all AUCs > 0.80, without the differences being statistically significant). The in-hospital overall mortality was 3.65%, with predicted mortalities according to additive EuroSCORE, logistic EuroSCORE and EuroSCORE II of 5.14%, 6.60% and 3.51%, respectively. Observed to expected (O/E) mortality ratio confirmed good calibration for entire cohort only for EuroSCORE II (1.05, 95% confidence interval 0.80-1.30). Hosmer-Lemeshow test confirmed overall good calibration only for additive EuroSCORE (p = 0.129). The EuroSCORE II significantly overestimates perioperative risk only in a low risk category (predicted mortality 1.29%, observed 0.7%). Affiliation to the higher EuroSCORE II risk group also denoted significantly longer period of stay in the intensive care unit, and significantly prolonged postoperative stay in the hospital (Friedman test, p < 0.001).

## Discussion/Conclusion

EuroSCORE II confirmed good discriminative capacity and good calibration ability (O/E mortality ratio) in a contemporary patients' cohort undergoing cardiac surgery at our institution.

